# Telepathology in cervical and breast cancer screening programmes

**DOI:** 10.1186/1746-1596-8-S1-S28

**Published:** 2013-09-30

**Authors:** Romano Colombari, Roberto Mencarelli, Alessio Gasparetto, Carla Cogo, Antonio Rizzo

**Affiliations:** 1 Anatomic Pathology, Fracastoro Hospital, ULSS 20, Verona, Italy; 2 Clinical Pathology Department, Local Healthcare Authority, Rovigo, Italy; 3 Information Technology Department Local Healthcare Authority, Rovigo, Italy; 4 Cancer Registry Veneto Region, Italy; 5 Anatomic Pathology, S. James Hospital, ULSS 8, Castelfranco Veneto (Tv), Italy

## Background

This study is about the application of telepathology in external quality assurance (EQA) for cervical and breast cancer screening programmes.

In Veneto Region, Italy, an organized effective mass-screening programme for cervical and breast cancer has been active since 1998. The previously experienced EQA with slide set circulation on a voluntary base by part of the Anatomic Pathology laboratories of this Region had failed because of several complications: slide’s circulation around more than 20 Anatomic Pathology services takes a great deal of time; there is a high risk of slide loss and/or break down during the multiple slides’ deliveries; patients’ privacy and operators’ anonymity can be regrettably compromised.

The EQA has recently become a mandatory requirement for accreditation of laboratories providing screening services within Veneto.

The whole image acquisition technology of cytological and histological slides, thank to innovative software, has become available in the last few years. These applications permit pathologists to storage digital images and to examine, on digital screen, virtual slides scanned in a far away laboratory, using a viewer downloaded from internet in an easy and, in most cases, free way.

In 2008 a slide scanner (Aperio ScanScope-XT) was installed at Anatomic Pathology Laboratory in Rovigo, Italy. This technical tool, with the proper ICT (Information and Communication Technology) configurations and architecture, became available to the Anatomic Pathology Units in Veneto.

Such technology is still in early application stage, so that we evaluate its potential use in EQA for cervical and breast cancer screening programmes.

## Material and methods

In 2008 a committee (one for cervical and one for breast cancer screening) has been elected among the pathologists involved in Veneto mass-screening programme for cervical and breast cancer to plan the EQA.

From 2009 to 2011 a year topic has been chosen among those hot in the field of cytopathology and histopathology of cervical and breast cancer screening.

Every year, in Veneto each laboratory participating in the project selected from routine files 1 to 3 cervical and breast cytologic and histologic samples. The selected samples were mailed to Clinical Pathology Department in Rovigo, where they have been anonymized and digitalized by using a ScanScope-XT. The images have been stored on a virtual slide repository available online for a web consultation. The personalized free access has been made available on website [1]. The experience with virtual slide technical tools was very different among the participant pathologists. No training, focused on virtual pathology, was given to the participant pathologists before the virtual slide-based EQA project started. Remote ICT support, when requested, was available to each participant. The project manager of each screening group collected the anonymized diagnoses. Each committee provided a gold standard diagnosis. The discordant cases have been proposed for plenary discussion in a year meeting, in the attempt to reach agreement among the participants.

## Results

Twenty-three public laboratories out of twenty-four active in Veneto joined the project. From 2009 to 2011, virtual slides have been created from 98 cervical smears and from the 102 corresponding biopsies. Similarly, virtual slides have been created from 52 breast cancer samples, 48 breast needle core biopsies and 95 breast FNAC. On virtual cervical slides, a total of 1717 cytological diagnoses and 1719 histopathological diagnoses have been obtained; on virtual breast slides, a total of 1717 cytological diagnoses and 1822 histopathological diagnoses have been obtained.

In 2009 only 59% of the participant laboratories for cervical EQA and 80% for breast EQA could successfully evaluate the virtual slides. Most of the participants couldn’t download and install the viewer or complained the low performances during slides’ evaluation.

In 2009 some non-sense incoherent diagnoses have been registered.

In 2011 the virtual slides have been successfully evaluated by all participants.

## Discussion

EQA has become an integral part of mass-screening programme development in Italy. Ideally, the scheme should include slide set circulation, but several obstacles prevent the adequate diffusion of such practice. The EQA based on circulating slides is still practiced but in only a few “niche” applications [2]. For this reason, the use of digital slides would represent a helpful alternative for the EQA [3,4].

The major issues limiting the use of virtual slide-based EQA in the first years of our EQA project did not involve image acquisition or quality but rather the pathologist's experience with virtual slide technical image management and several issues such as the pathologist's interface and the hospital's network. The need for standardization of technical elements of image has already been pointed out [5,6]. Moreover, very recently these technical standards in the contest of mass-screening programme have been established by a preeminent European committee [7].

Several challenges can be pointed for the next few years to allow the wide and easy application of virtual pathology in EQA: on one hand there is the need to focus on pathologist's training with virtual slide technical tools; on the other hand, there is the need to improve the hospital's network, to homogenize the security policies, to adequate the technical tools of each Anatomic Pathology Unit to the recently proposed standard [7].

These results are encouraging to purse this workgroup, able to involve the participants in screening programmes, with a very good cost/benefit ratio.

## List of abbreviations

EQA: external quality assurance; ICT: information and communication technology

## Competing interests

The Authors declare that they have no competing interests.

## Authors’ contributions

RC and AR: scientific coordinators and project managers

RM: virtual slide quality supervisor

AG: ICT support

CC: continuing medical education regional coordinator

Veneto cervical cancer screening group: cervical screening committee

Veneto breast cancer screening group: breast screening committee

Veneto cervical cancer screening group: Romano Colombari and Enzo Bianchini, Laura Borghi, Loredana Bozzola, Concetta Chiarelli, Duilio Della Libera, Licia Laurino, Lorella Marchioro, Barbara Pertoldi, Antonella Pinarello, Paola Rossi.

Veneto breast cancer screening group: Antonio Rizzo and Enzo Bianchini, Laura Borghi, Roberta Boschetto, Giuseppe Briani, Andrea Caneva, Elisa Canova, Giovanni Capitanio, Duilio Della Libera, Stefania Dante, Orfeo Del Maschio, Roberto Di Pietro, PierPaola Gasparini, Licia Laurino, Genesio Leo, Gigliola Ludovichetti, Paolo Machin, Lorella Marchioro, Marcello Lo Mele, Enrico Orvieto, Salvatore Paolino, Ida Pavon, Antonella Pinarello, Quirino Piubello, Domenico Reale, Daniela Reghellin, Vittorio Rucco, Debora Tormen.
